# An Effective Image-Based Tomato Leaf Disease Segmentation Method Using MC-UNet

**DOI:** 10.34133/plantphenomics.0049

**Published:** 2023-05-15

**Authors:** Yubao Deng, Haoran Xi, Guoxiong Zhou, Aibin Chen, Yanfeng Wang, Liujun Li, Yahui Hu

**Affiliations:** ^1^ College of Computer & Information Engineering, Central South University of Forestry and Technology, Changsha, 410004, Hunan, China.; ^2^ College of Mechanical & Electrical Engineering, Central South University of Forestry and Technology, Changsha, 410004, Hunan, China.; ^3^ National University of Defense Technology, 410015, Changsha, Hunan, China.; ^4^ Department of Soil and Water Systems, University of Idaho, Moscow, ID, 83844, USA.; ^5^ Plant Protection Research Institute, Academy of Agricultural Sciences, 410125, Changsha, Hunan, China.

## Abstract

Tomato disease control is an urgent requirement in the field of intellectual agriculture, and one of the keys to it is quantitative identification and precise segmentation of tomato leaf diseases. Some diseased areas on tomato leaves are tiny and may go unnoticed during segmentation. Blurred edge also makes the segmentation accuracy poor. Based on UNet, we propose an effective image-based tomato leaf disease segmentation method called Cross-layer Attention Fusion Mechanism combined with Multi-scale Convolution Module (MC-UNet). First, a Multi-scale Convolution Module is proposed. This module obtains multiscale information about tomato disease by employing 3 convolution kernels of different sizes, and it highlights the edge feature information of tomato disease using the Squeeze-and-Excitation Module. Second, a Cross-layer Attention Fusion Mechanism is proposed. This mechanism highlights tomato leaf disease locations via gating structure and fusion operation. Then, we employ SoftPool rather than MaxPool to retain valid information on tomato leaves. Finally, we use the SeLU function appropriately to avoid network neuron dropout. We compared MC-UNet to the existing segmentation network on our self-built tomato leaf disease segmentation dataset and MC-UNet achieved 91.32% accuracy and 6.67M parameters. Our method achieves good results for tomato leaf disease segmentation, which demonstrates the effectiveness of the proposed methods.

## Introduction

Tomato is native to South America and is a major food crop in temperate and tropical climates due to its great culinary and medicinal value [1]. It may be grown in any well-drained soil type. As a result, tomatoes may be seen in a variety of environments, from tiny home gardens to large-scale planting, and they are now extensively grown across the world. Tomato, like other crops, is susceptible to a range of pests and diseases as they grow. Similar to diseases that affect tomato fruits, tomato leaves are also susceptible to disease [2]. If not handled promptly, it will decrease production and even crop failure [3]. Previously, people subjectively identified the type of tomato disease based on experience [4]. However, such methods had poor discriminating capacity, cannot recognize reliably, and are laborious [5]. Image processing technology has been developed over a long period and is now being used in a wide range of industries, including agriculture. It is simply necessary to gather and preprocess disease image samples, then extract the features of the disease area in the image using a feature extraction method, and finally input the feature information into the model for training [6]. However, as agricultural modernization progresses toward intelligence, traditional image recognition algorithms will be unable to cope with the complicated reality [7–9]. Timely and precise identification of tomato leaf diseases, as well as the accompanying control measures, are essential for maintaining crop production and increasing revenue [10,11].

With the improvement of the computer performance, applying deep learning to agricultural production is the development trend of agriculture in the future[12,13]. Deep learning automatically extracts image features by introducing operations such as convolution layer, pooling layer, and full connection layer, which makes a breakthrough in plant leaf disease recognition. At present, there are 2 main methods: region detection based on bounding box and segmentation based on semantics [14]. Detection focuses on the proposed area for locating the disease, while segmentation is the binary classification of pixels in the image to distinguish the edges of actual and nonactual target areas. Judging from the labeling time, the detection bounding box takes less, but it is difficult to accurately describe the actual area of the target. With the development of intellectual agriculture, we need more elaborate leaf disease control measures. Although the production of segmentation labels is more time-consuming, accurate segmentation results can quantify the specific area of the disease in space. On this basis, we can evaluate the severity of the disease and take different measures to control it.

**Fig. 1.**
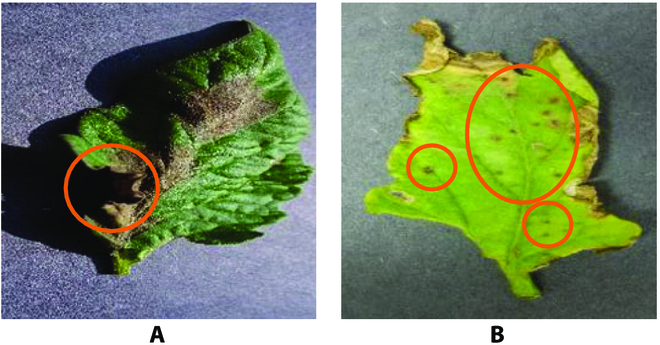
Demonstration of tomato leaf disease characteristics. In (A), the edge details of the mark site are blurred due to lighting. In (B), the disease in the marked part is too small.

We tried to use UNet to segment plant leaf diseases. The network performs well in common diseases but poorly in 2 aspects: (a) tomato leaf image has the problem of blurred edges. As shown in Fig. 1A, the edge details of the mark site are blurred due to lighting. As a result, the features of edge diseases are too small to be captured effectively by conventional segmentation networks. Furthermore, it seriously affects the segmentation effect of the network and even results in completely wrong segmentation results. (b) Some small tomato leaf diseases are easy to be ignored. As shown in Fig. 1B, the disease in the marked part is too small. This results in the loss of small but critical feature information, causing the model to ignore the disease region.

To address the edge blurring problem, Yuan et al. [15] optimized the model segmentation branch using an attention mechanism and a 2-layer 3 × 3 convolution module to improve the edge feature representation as well as used a multiscale blade segmentation strategy during model testing, utilizing the optimal targets on multiple scales. Ji et al. [16] used median filtering and image sharpening to process the leaf images in order to preserve the disease areas and edge details of the images as much as possible. Considering that the unique concatenation structure of UNet effectively integrates the different channel characteristics of the disease, we designed a multi-scale convolution module. This structure increases the channel dimension of single-layer feature map and makes the model contain more high-level and low-level feature information. It is helpful to feel the edge characteristics of leaf disease.

To address the problem of tiny leaf diseases, Cheng et al. [17] developed a perceptual organization model that captures the noncontingent structural relationships between structured object components so that they can be grouped accordingly. Wang et al. [18] developed a new multiscale Transformer module to further improve the model's ability in extracting multiscale long-dependent global features of retinal lesions in optical coherence tomography images. Based on the concatenation structure of UNet, we propose a cross-layer attention fusion mechanism. This mechanism can model different channel features, highlighting differences between tiny diseases and healthy areas. It is placed in each decoding layer to integrate the diseases-characteristic information of each layer. This facilitates the model to feel the edge characteristics of the leaf disease.

In this paper, an MC-UNet module is proposed for the segmentation of tomato leaf diseases. The contributions are summarized as follows:

1. A Multi-scale Convolution Module (MCM) structure is proposed. By using convolution kernels of different sizes to extract spatial information of tomato leaves of different scales, the problem of partial scale information loss caused by traditional convolution is solved. Squeeze-and-Excitation Module (SE Module) is also used to emphasize the disease feature image areas so that the edge of the disease can be expressed more accurately.

2. A Cross-layer Attention Fusion Mechanism (CAFM) is proposed. In order to suppress irrelevant information and focus on tomato features, the gating structure is introduced. We replace the output layer of the network with CAFM, which further fuses the information of tomato features at each level.

3. The SoftPool function was chosen for pooling. SoftPool uses the Softmax weighting method. Compared with the original MaxPool method, SoftPool can retain more disease feature information.

4. The SeLU activation function was selectively used in the network. Compared with the rectified linear unit (ReLU) activation function, the SeLU function can make the sample distribution satisfy zero mean and unit variance and avoid the neuron death phenomenon.

Since traditional plant disease detection is time-consuming and labor-intensive, scholars have been seeking to solve this problem using modern techniques. Segmentation methods for images can be divided into traditional feature extraction-based segmentation methods and deep convolutional neural network (CNN)-based image segmentation methods.

The segmentation method based on feature extraction has achieved good segmentation effect in different areas of image segmentation. Many scholars have proposed many improved segmentation algorithms on this basis. Yuan et al. [19] proposed a cucumber leaf disease image segmentation method under complex background based on red-green-blue color space and realized the segmentation task of different cucumber leaf diseases with this method. Wu et al. [20] solved the segmentation problem of maize damaged leaves by combining linear iterative clustering algorithm with regional growth. Lin et al. [21] used the method combining block threshold and edge detection to segment plant leaf images, used the improved Sobel edge operator to detect the edge of the lesion region in the segmented images, and spliced the detected subimages to get the whole diseased leaf image. Kaneko et al. [22] combined different feature extraction algorithms to complete the extraction of different features such as color, texture, and shape of diseased leaves in the feature extraction stage and then used the trained classifier to perform pixel classification on these feature maps to complete the segmentation of diseased leaves. These methods have great limitations, and the algorithm is highly complex, so it is difficult to apply in the actual agricultural production field.

Deep learning technology has recently enabled us to diagnose plant diseases based on images, providing new ideas for disease control [23]. It can identify disease areas by extracting features from a large number of labeled images. Liu et al. [24] coupled Markov with CNNs to complete picture segmentation of cotton spots. This method optimally trains CNNs by constructing a conditional random field energy function. Xiong et al. [25] completed the segmentation of rice ears at different growth stages using a combination of super pixel segmentation and CNNs. However, it performs poorly in complicated backgrounds. Ma et al. [26] established a CNN-based disease diagnosis system for greenhouse cucumbers. They combine composite color characteristics with CNNs in the preprocessing step to achieve complete disease segmentation of different categories. Pound et al. [27] employed CNNs to separate distinct parts of wheat root tips, wheat ears, and so on. The approach is difficult and not universal since this method involves preprocessing of the original image. The deep learning-based segmentation approaches mentioned above have been applied to many segmentation tasks. However, they have drawbacks such as single segmentation content, high network complexity, and long model training time. Zhao et al. [28] improved the CNN with deconvolutions to solve the problem of picture resolution degradation caused by upsampling. In the segmentation of grape disease leaves, their approach provides greater segmentation accuracy than the classic conditional random fields segmentation algorithm. However, training the model takes more time. Ren et al. [29] employed fully convolutional networks to conduct a segmentation experiment on tomato leaf disease spots since the leaf pictures of maize disease in the field are frequently impacted by changing lighting during the segmentation process. The DGVGGNet model outperforms the others, with pixel accuracy and average intersection ratio of disease segmentation achieving 94.66% and 0.7536, respectively. Chowdhury et al. [30] developed a model called EfficientNet. They only fine-tuned and trained the developed model to detect unhealthy tomato leaf images. The authors of this study mainly focus on the major diseases areas in the picture and are unable to detect tiny disease. Liang et al. [31] proposed an image-based network for plant disease detection and severity estimate (PD2SENet) with a 91% accuracy in predicting disease severity. Xiong et al. [32] proposed a grab-cut approach for extracting leaf areas. They discuss the automatic image segmentation algorithm, which combines the grab-cut approach and Mobile Convolutional Networks in the paper. The network achieves an average performance of 84% via this approach. However, the procedure of putting foreground and background markers during the grab-cut approach, on the other hand, is difficult and requires skilled interaction.

The UNet network is a well-known deep learning algorithm for image segmentation. It uses deep architectures to automatically extract features from input data [33]. The multilayer structure enables the learning of more abstract characteristics and categorization at the pixel level. Because of its remarkable performance, the architecture was commonly utilized when it was proposed, and many academics chose it as a baseline to integrate with their work. Wang et al. [34] proposed the Efficient Non-local Residual U-shape Network (ENRU-Net), which improves the nonlocal blocks with a U-shaped network structure to capture contextual information. Xu et al. [35] enhanced UNet by introducing an attention mechanism and proposing a new loss function. They outperformed UNet in terms of F1 score by more than 10.78%. Extensive studies by Liu et al. [36] demonstrated that combining the pyramid scene and residual connections can enhance UNet’s capacity to capture global background information. Zhang et al. [37] use deep separable convolution to improve UNet and design and apply and demonstrate the effect of XU-Net. Zhou et al. [38] introduced encoder and decoder subnetworks and connected them together via stacked and dense skip connections. Their studies prove that the network overcomes the drawbacks of skip connections and employs deep supervision to assist in choosing a path on its own, and the network performs excellently. Other improvements include combining UNet and Mask R-CNN as an ensemble model and embedding a pretrained VGG encoder in UNet to enhance UNet extraction capacity [39,40].

In this work, although the network performs well on common leaf diseases (such as distinguishing features and regular size), it does not perform well for cases with blurred edges and tiny diseases. Inspired by the above researchers and combined with the characteristics of the above-mentioned diseases and UNet, we designed MC-UNet to solve these problems.

## Materials and Methods

### Data acquisition and expansion

The majority of the tomato leaf disease images used in this study come from the PlantVillage dataset [41]. It is a public database in which tomato disease images may be classified into 9 categories. Because the number of data labels was unevenly distributed, 4 sample disease tomato categories were chosen: Bacterial spot, Late blight, Early blight, and Leaf mold. We sifted through the dataset. There are additional images from other websites that we collected with the assistance of specialists. Finally, 4,622 disease leaves were collected.

**Fig. 2.**
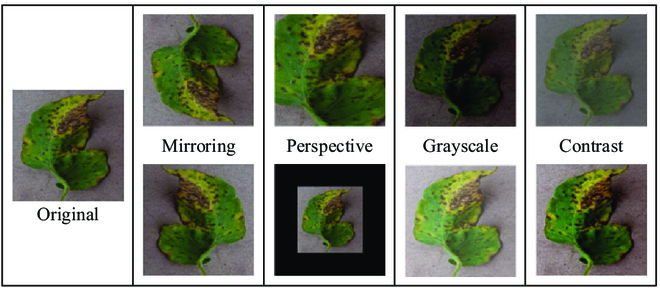
Eight transformation images of bacterial spot.

The training of segmentation models requires a large number of samples, while acquiring a large number of disease images is a considerable challenge. Therefore, we employed the processes of (a) mirroring, including up-down mirroring and left-right mirroring; (b) perspective, including a 70% size and 130% size adjustment; (c) adjusting the grayscale, including adjusting to 80% and 120% of the original grayscale; (d) adjusting the contrast, including adjusting to the original contrast 70% and 130%. Figure 2 shows augmented images of a disease image of a Bacterial spot. Mirroring and perspective can help to improve the geographic distribution of illnesses. The transformation of grayscale and contrast can simulate the feature information of a variety of diseases, making the segmentation of tomato leaf diseases in complex environments more accurate. Table 1 shows the number distribution and images of 4 different tomato-diseased leaves.

**Table 1.** Number and proportion of tomato leaf images.

Category	Example	Number (before)	Number (after)	Proportion/% (after)
Bacterial spot	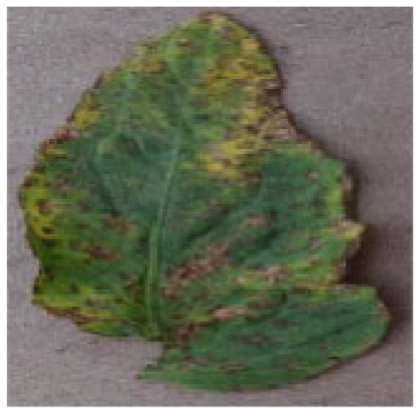	1,032	1,595	25.03%
Late blight	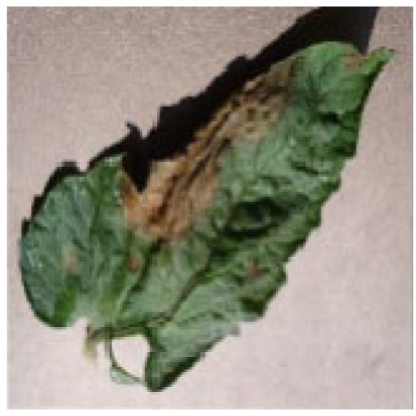	1,238	1,488	23.35%
Early blight	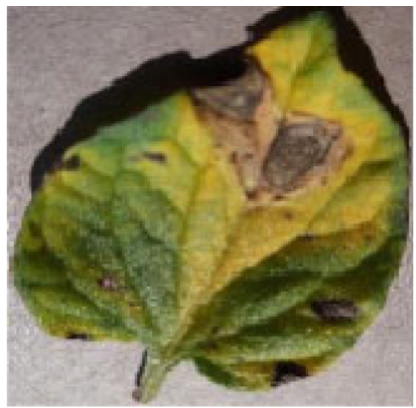	1,000	1,688	26.50%
Leaf mold	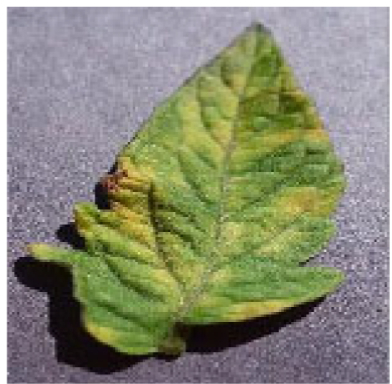	952	1,601	25.12%

**Fig. 3.**
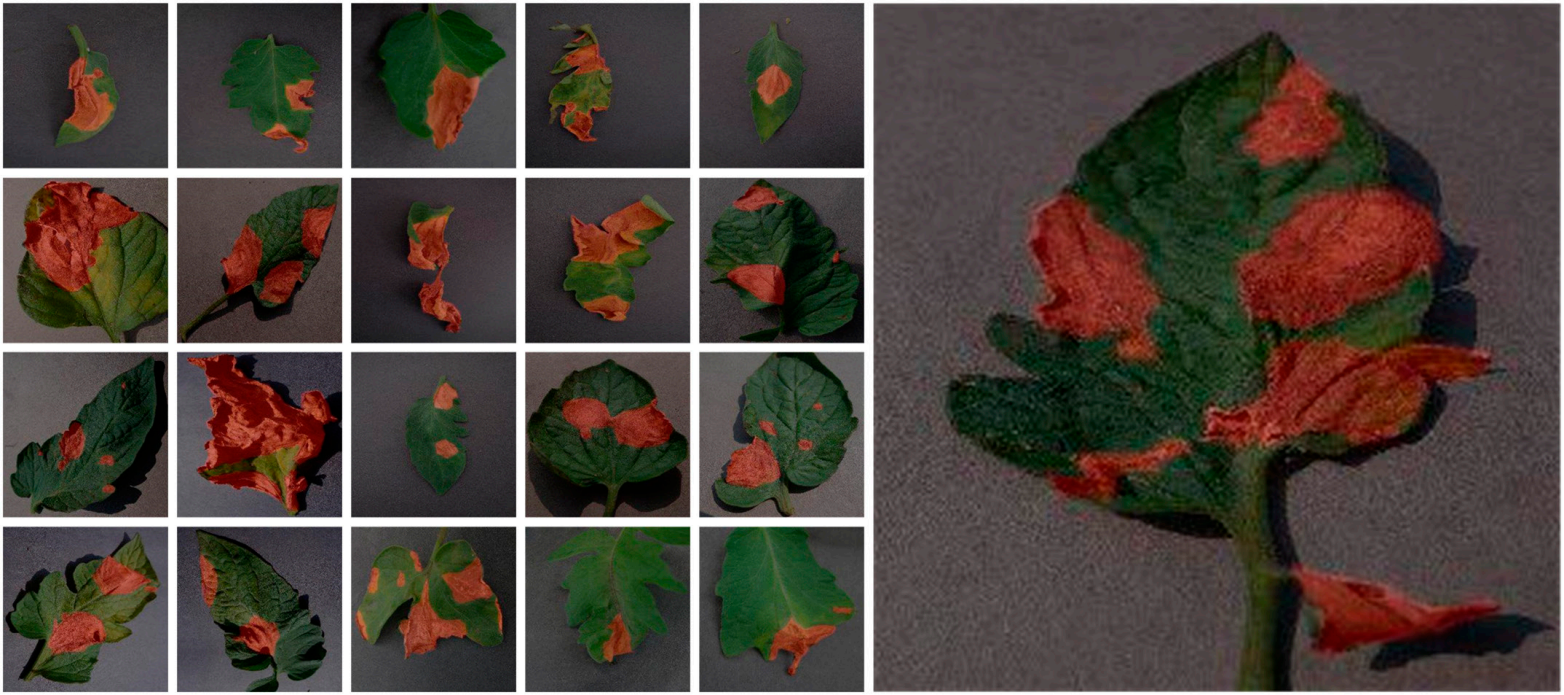
Presentation of some sample labels.

The images size is uniformly adjusted to 256 × 256 for research. We use Labelme software to mark tomato disease sites and generate mask maps. Each image’s annotations are saved in json format. Figure 3 shows a partially annotated image sample, with unhealthy areas highlighted in red.

### MC-UNet

**Fig. 4.**
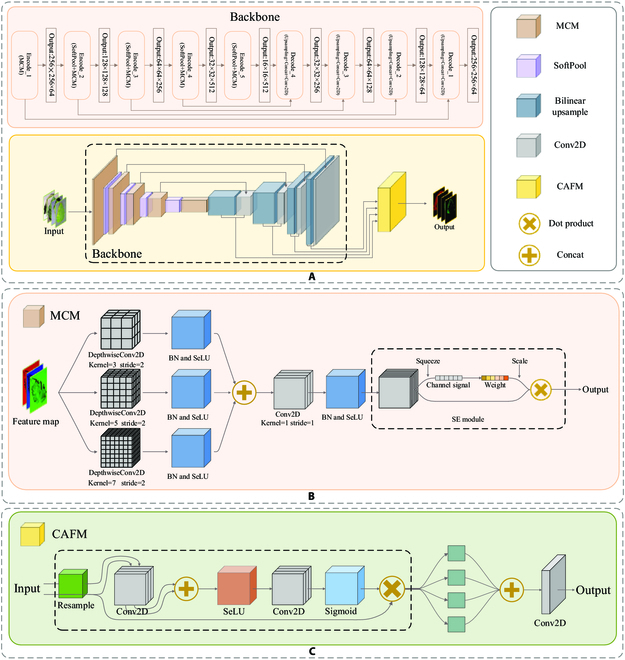
MC-UNet structure diagram. (A) The overall structure of MC-Unet. (B) The multiscale convolution module. (C) The cross-layer attention fusion mechanism.

We propose an MC-UNet model to address the mentioned problems. Figure 4 shows the overall design of MC-UNet. The network’s encoder employs SoftPool and MCM, which halves the picture size and doubles the number of feature maps, respectively. The decoder consists of 2 parts: bilinear upsampling, which doubles the size of feature maps, and convolution blocks, which reduce the number of feature maps in half. Finally, we input each different scale image into CAFM to fuse the feature information in different dimensions. Each module will be thoroughly explained below.

#### Multiscale Convolution Module (MCM)

Convolution is a fundamental neural network operation that can filter the features in the receptive field and extract the most prominent characteristics from the region. However, convolution with a single scale only learns limited features, while accurate segmentation requires distinct tomato edge features. Multiscale Convolution Module (MCM) is proposed for blurred images of tomato disease that may maintain image information while conducting standard convolution operations. Figure 4B shows its structure. We split the input into 3 branches for different operations. Because less computation is conducted in depthwise convolution, larger convolution kernels may be employed to broaden the receptive field. So, on the 3 branches, we employ the convolution kernels 3 × 3, 5 × 5, and 7 × 7 to conduct operations that more fully reflect the disease features. The outputs of the 3 branches are then fused to enhance the expression of disease characteristic information. Finally, a 1 × 1 convolution is utilized to compress the channel, decrease the dimension, and merge the multiscale tomato disease feature information extracted by the 3 channels. We introduce the SE Module [42] to ensure that the module can pay more attention to the disease’s edge characteristics. This boosts the prominence of the disease feature map’s edge region and lowers interference caused by redundant information, such as blurring of the illness image. The SE Module is a channel-based attention mechanism with 2 stages: squeeze and excitation.

The compression stage involves the following steps: First, apply global average pooling on the feature map of the input *w* × *h* × *c* to construct a feature map with a global receptive field and a size of 1 × 1 × *c*. Equation 1 explains the feature map computation procedure for the *i*th channel:

$$z_{i}=\frac{1}{w\times h}\sum_{p=1}^{W}\sum_{q=1}^{H}u_{i}\left(p,q\right)$$ (1)

In the formula, *w* × *h* represents the original feature map resolution; *u_i_*(*p*, *q*) represents the element whose *i*th channel layer coordinate is (*p*, *q*), and the total number of channels is *C*; *z_i_* is the feature map amount of this channel. A 1 × 1 × *C* vector *z* ∈ *ℝ^C^* is generated through this compression method. Equation 2 depicts the stimulation process:

$$s=\sigma \left(g\left(z,W\right)\right)=\sigma \left(W_{2}\delta \left(W_{1}z\right)\right)$$ (2)

First, we reduce the channel dimension to *C*/*r* by a point-by-point convolution layer with weight *W*_1_, where the *r* channel dimension is reduced by a factor of *r*. Next, we pass a point-by-point convolution with weight *W*_2_. Then, the channel dimension is recovered after activation using the SeLU(*δ*) function. Finally, the Sigmoid(*σ*) function is used to generate the normalized channel weights *s* ∈ *ℝ^C^* with a scale of 1 × 1 × *C*. The normalized channel weights are multiplied with the corresponding channels in the original feature map to obtain the channel attention feature map.

The model will be able to combine tomato disease feature information from various sources and generate related feature maps by utilizing MCM. The effectiveness of MCM presents an experimental evaluation of MCM as well as experimental results comparing MCM to other attention mechanisms.

#### Cross-layer Attention Fusion Module (CAFM)

Some tomato leaves have tiny disease. The traditional model is ineffective of extracting such conditions, which affects the segmentation results. The attention mechanism is inspired by the human visual process, and its core is to extract specific characteristics more precisely in the neural network using a series of attention weight distribution coefficients [43]. Low-level feature maps contain more location information, while high-level feature maps contain abundant feature information. In order to make good use of limited visual information processing resources, networks need to focus attention on specific areas. This reduces the network's dependence on external information and automatically responds to target areas when the network has not yet generated an area proposal. In addition, the attention mechanism simulates the position relationship of global features, so that similar features can enhance each other and thus improve model sensitivity and accuracy. However, the feature information capturing capability of the regular attention mechanism is weak, and it can only perform well with large datasets. Therefore, the gating structure is introduced to correct the deep feature maps generated during the encoding process. This preserves location information and the characteristics of the target. Therefore, it is commonly utilized for tasks like classification [44], machine translation [45], image captioning [46], etc. We construct a Cross-layer Attention Fusion Module (CAFM) with a gating structure in this paper and use different weight assignment methods for disease features and nondisease features to focus learning on tiny disease features. Figure 4C shows the structure. The module improves the disease image’s feature expression, helping the model to acquire more accurate and full disease feature information, and reducing the impact of irrelevant regions. The final classification result image contains multiple-scale information, which can improve the model’s multiscale generalization ability. The gating structure is divided into 3 sections:

1. Feature extraction. As shown in Eqs. 3 and 4), mean pooling is used to construct the weight matrices *W_x_* and *W_g_* of the feature map that contain *C* channel information. *g* and *x* are the decoding map matrix and the encoding map matrix, respectively, *H* and *W* are the height and width of the feature map, *C* is the number of channels, and *W_x_* and *W_g_* are the feature weight matrices.

$$W_{x}=\frac{1}{H\times W}\sum_{i=0}^{H}\sum_{j=0}^{W}x\left(i,j\right)$$ (3)

$$W_{g}=\frac{1}{H\times W}\sum_{i=0}^{H}\sum_{j=0}^{W}g\left(i,j\right)$$ (4)

2. Feature weight update. In Eq. 5, *σ*_1_ represents the ReLU activation function, and *σ*_2_ represents the sigmod activation function. By dot product process, the fully concatenated operation of simultaneously encoding and decoding portions of the feature map can reduce the amount of parameter calculation; The outputs of the fully connected layer are then summed together and transmitted through the ReLU layer, where the result is multiplied by the point Φ to establish a full connection to yield *q_att_*, which is the intermediate matrix. Equation 6 indicates that the intermediate result is then subjected to the *σ*_2_ activation function, where *θ_att_* represents a group of parameters that includes *W_x_*, *W_g_*, and Φ. *W_x_* and *W_g_* complete the weight backpropagation learning and the updating of *g* and *x* feature weights by the above operations. Finally, the updated weights are mapped to the feature maps.

$$q_{\mathrm{att}}=\Phi^{T}\left(\sigma_{1}\left(W_{x}^{T}x+W_{g}^{T}g\right)\right)$$ (5)

$$\alpha =\sigma_{2}\left(q_{\mathrm{att}}\left(x;\theta_{\mathrm{att}}\right)\right)$$ (6)

3. Feature map update. The dot product of the feature map *x* and the updated weight matrix *α* is represented in Eq. 7. The weights attributed to other backgrounds are now decreased. The output, or the attention mechanism feature map, is obtained, and its steps are linked to the decoding network for upsampling.

Output=x×α (7)

Finally, the corresponding feature map may be formed by fusing and convolving the gated output. We utilize the output of bilinear upsample as the gate terminal (corresponding to the top of Input) to emphasize the features of mild diseases. The gated feature map is generated after emphasizing the feature variance by feature fusion and an activation function. The gated result is obtained by dot-multiplying the gated feature map with the original convolution output. In the figure, 4 couples of Input correspond to the 4 gated outputs. Finally, they are fused and convolved, preserving the more comprehensive and finer feature information. The validation experiments of the CAFM mechanism’s efficacy are described in Effectiveness of CAFM.

#### SoftPool

The pooling layer in the CNN plays a role in lowering the size of the feature map, so feature information loss should be minimized during the pooling process. MaxPool is used for feature extraction in traditional UNet. During the pooling process, MaxPool selects the maximum activation value of the feature map while ignoring other associated information. It is unable to maintain feature information in some sick images with inconspicuous features. In address to this problem, this study employs SoftPool in place of the original MaxPool. By using the Softmax weighting approach, SoftPool maps each pixel in the visual field to the next layer of the network. This pooling method, as compared to MaxPool, can maintain more disease feature information in the feature map when downsampling the feature map.

**Fig. 5.**
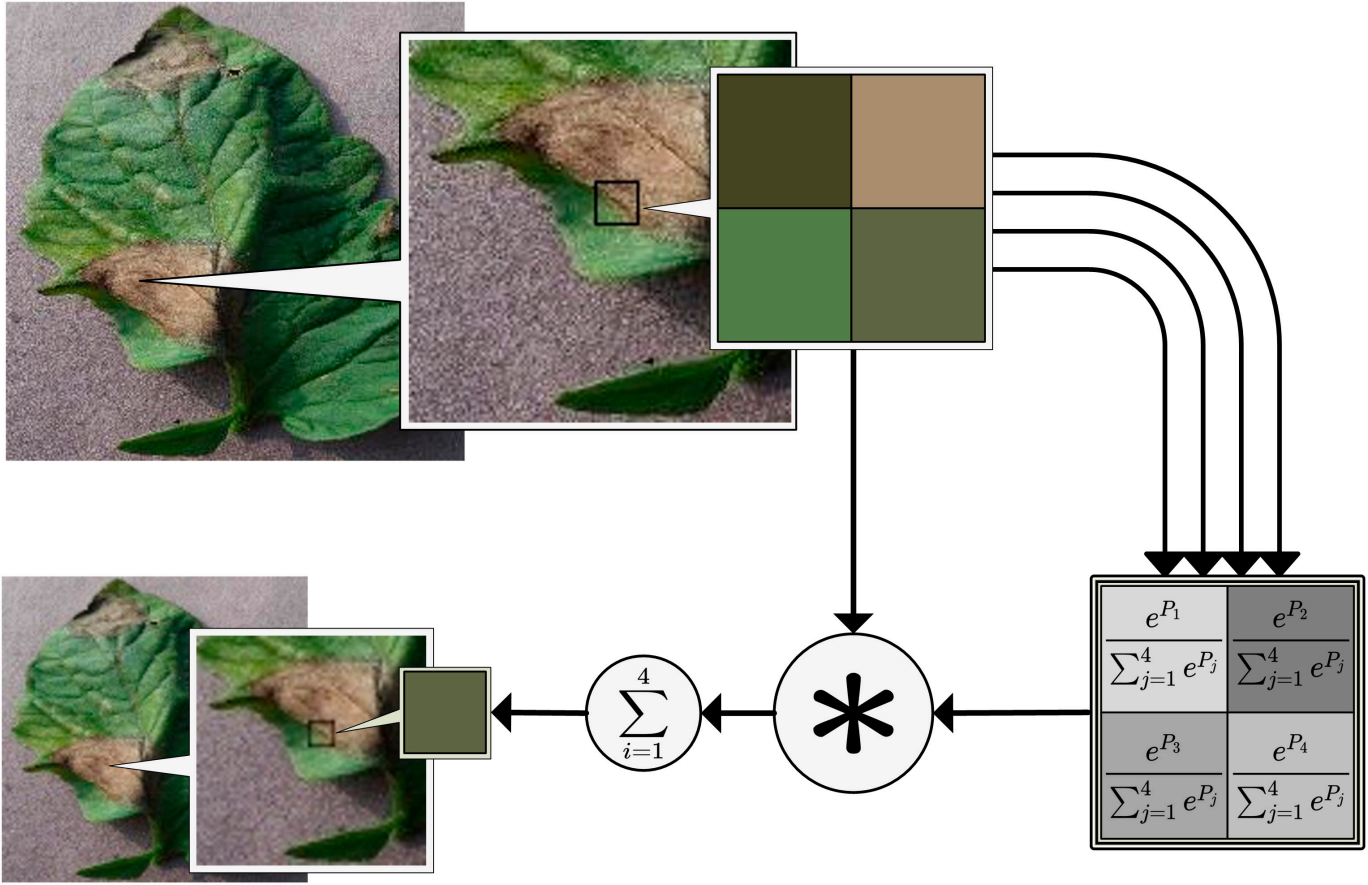
SoftPool exponential weighting process.

Figure 5 depicts the SoftPool operation. The function uses the natural exponent *e* as the base to ensure that the activation value is proportionate to the related output effect. This process is differentiable, which means that during backpropagation, all activations inside the local kernel neighborhood will be assigned at least 1 minimum gradient value. Each activation factor is allocated a distinct weight inside the activation zone, and each activation *a_i_* is assigned a weight *ω_i_*. The weight *ω_i_* is calculated by dividing the natural exponent of the activation by the total of the natural exponents of all activations in the activation region: Each activation *a_i_* in the activation region is given a weight *ω_i_*. The weight *ω_i_* is the activation’s natural index divided by the sum of all activations’ natural indices in the activation region.

$$\omega_{i}=\frac{e^{a_{i}}}{\sum_{j\in R}e^{a_{j}}}$$ (8)

The SoftPool method’s output value is the weighted sum of all activations in the activation region:

$$\bar{a}=\sum_{i\in R}{w_{i}}^{*}a_{i}$$ (9)

#### SeLU activate function

MC-UNet employs SeLU as the activation function, to ensure stable training. The generator’s activation function in UNet is ReLU. Equation 10 is the ReLU activation function. According to it, the gradient is very small since the derivative of its function on the positive semiaxis is always 1, so the gradient can quickly disappear while passing through the function. Furthermore, the ReLU function is set to zero on the negative semiaxis, resulting in neuron death when the negative gradient passes through the ReLU unit.

The positive semiaxis derivative of the SeLU activation function is higher than 1. Equation 11 is the SeLU activation function. Therefore, when the variance is insufficient, it may be increased without causing the gradient to vanish. To overcome the problem of neuron death, the negative semiaxis is no longer directly set to zero. Because its slope is rather soft, the network selects SeLU as the activation function to maximize the unilateral inhibition advantage of the activation function.

ReLU=x,ifx>00,ifx≤0 (10)

$$\mathrm{SeLU}=\lambda \left\{\begin{array}{c} x,\mathrm{if}\;x>0\\ {\mathrm{ae}}^{m},\mathrm{if}\;x\leq 0 \end{array}\right.$$ (11)

**Fig. 6.**
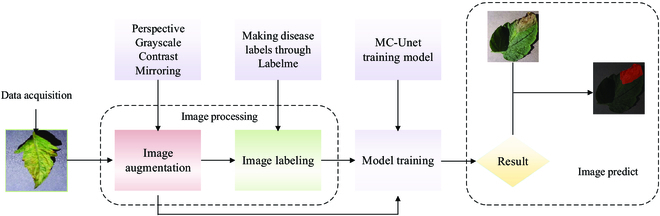
The system building process diagram of our proposed MC-UNet.

Benefit from specific design of each module for blurred edge and tiny disease, MC-UNet achieves good result in the segmentation of tomato leaf disease. Our model can be deployed on professional detection equipment to construct an automatic tomato leaf disease assessment system. The diagram of the system built based on MC-UNet is shown in Fig. 6.

## Results

This section is divided into 4 sections: (a) preparation of the experimental environment and hyperparameter setting; (b) preparation of the experimental dataset; (c) definition of the parameters and evaluation of the effectiveness of each module; (d) comparison of MC-UNet with other methods, which demonstrates that MC-UNet has better results in tomato disease segmentation.

### Experiment setting

This work’s experiments are all conducted on the same hardware and software platform. Table 2 shows the hardware configurations employed for training and testing. Because the best hyperparameter combination depends not only on the model but also on the hardware and software environment, each network’s hyperparameters are uniformly designed to avoid the effect of hyperparameters on experimental results. Table 3 shows the hyperparameters that were determined after numerous tests.

**Table 2.** Hardware and software parameters.

Hardware environment	CPU	Intel(R)Xeon(R)CPU 2.00GHz
	GPU	NVIDIA Tesla V100-SXM2
	RAM	16GB
	Video memory	16GB
Software environment	OS	Ubuntu 18.04. 5
	CUDA Toolkit	V11.1
	CUDNN;	V8.0.4
	Python	3.8.8
	torch	1.2.0
	torchvision	0.4

**Table 3.** Experimental settings.

Size of input images	256 × 256
Batch	8
Initial learning rate	0.0001
Optimizer	Adam
Momentum	0.9

### Dataset processing

We performed data augmentation on the basis of 4,622 original disease images in Data acquisition and expansion. The final 6,372 images were obtained, and the number of images for each type of disease was similar. Cross validation, also known as rotation estimation, is a useful method for statistically dividing a data sample into smaller subgroups. First, the train set is used for training. Subsequently, the validation set is used to test the trained model and to evaluate the performance of the network. The images used for the validation set are all chosen from the original images for experimental purposes. We also put aside 10% of the images to test the trained network, which is known as the test set. We split the dataset based on the training set: validation set: test set ratio (8:1:1), which means that the training set has 5,098 tomato disease images, while the validation set and test set each contain 637 tomato disease images. Labelme software marked the training and test sets, and the corresponding disease mask map was generated.

### Evaluation indicators

We first introduce 4 different types of area definitions: True positives (*TP*) represent areas that have actual disease and are predicted to have disease. True negatives (*TN*) represent areas that are free of disease and are predicted to be free of disease. False positives (*FP*) represent no disease, but the area is predicted to be infected. False negatives (*FN*) represent there is a disease that is predicted to be healthy. Table 4 shows 4 regional definitions for segmentation results.

**Table 4.** Four regional definitions for segmentation results.

Labeled	Predicted	Regional definitions
Positive	Positive	*TP*
Positive	Negative	*FN*
Negative	Positive	*FP*
Negative	Negative	*TN*

We use several metrics of Accuracy, mean Average Precision (mAP), FPS, and Parameter size (Parameters) to assess the model’s efficacy thoroughly.

$$\mathrm{Accuracy}=\frac{\mathrm{TP}+\mathrm{TN}}{\mathrm{TP}+\mathrm{FP}+\mathrm{FN}+\mathrm{TN}}$$ (12)

$$\mathrm{Precision}\left(P\right)=\frac{\mathrm{TP}}{TP+\mathrm{FP}}$$ (13)

$$\mathrm{Recall}\left(R\right)=\frac{\mathrm{TP}}{\mathrm{TP}+\mathrm{FN}}$$ (14)

Precision is the ratio of correctly segmented tomato leaf images in the dataset that are correctly and wrongly segmented. Recall is the ratio of all tomato leaf images that are assumed to require segmentation to all tomato leaf images that truly require segmentation. We analyze the model comprehensively by combining the precision and recall and decide to utilize the average precision mAP as the evaluation metric:

$$\mathrm{mAP}=\int_{0}^{1}P\left(R\right)\mathrm{dR}$$ (15)

FPS denotes the number of pictures detected by the model per second (detection speed). *T* denotes the time necessary to test a single sample.

$$\mathrm{FPS}=\frac{1}{T}$$ (16)

Dice coefficient, also known as Sorensen-Dice coefficient, is a function that measures how similar 2 objects are. It is often used to compute the similarity of 2 samples as follows: (Label represents our personally labeled disease category, i. e. Ground Truth):

$$\mathrm{Dice}=\frac{\mathrm{TP}\cap \mathrm{Label}}{\mathrm{TP}\cup \mathrm{Label}}$$ (17)

The introduction of statistical tests enables to evaluate whether the method in this paper has a significant improvement in performance. In the mask maps, each pixel point is a binary variable (0 for no disease; 1 for disease). For a diseased image, the position codes of each pixel in the model output and the manually labeled mask map correspond. For all test images, their position codes are unique. Specifically, each location code is the independent variable, and the pixels in the model output and the manually labeled mask maps are the 2 sets of dependent variables. Since the locations where the diseases appear in the images do not show a certain regularity, this makes the values of the pixel variables not obey the normal distribution. Therefore, we used the Wilcoxon signed-rank method [47] to perform statistical tests.

### Experimental results and analysis

#### Comparison of different data augmentation methods

**Fig. 7.**
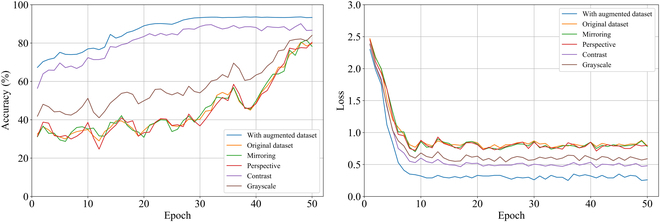
Comparison experiments of different data enhancement effects.

We evaluated the data augmentation methods with accuracy and loss, and a total of 6 sets of comparison experiments were conducted. Among them, 4 sets of experiments are single augmentation methods (Mirroring, Perspective, Contrast, or Grayscale), 1 set of experiments does not use data augmentation (Original dataset), and 1 set of experiments includes all augmentation methods (Augmentation dataset including Mirroring, Perspective, Contrast, and Grayscale); the result can be found in Fig. 7. The augmented data improve the performance of the network when compared to the original data. This is because images provide more feature learning opportunities for the network. In the figure, it can be seen that the mirroring image and perspective augmentation methods do not differ significantly from the original data in terms of accuracy. This is because these 2 augmentation methods did not change the leaf disease characteristics. However, the grayscale and contrast augmentation methods significantly improved the accuracy. This indicates that adjusting the grayscale and contrast is more helpful for model training, because higher accuracy indicates that the network’s generalization capacity is stronger. The loss value represents the gap between the model’s prediction results for the training set and the actual results. The faster the loss value decreases, the faster the model converges, and the stronger the network segmentation ability. In summary, we augment the dataset with the ratio of Mirroring: Perspective: Grayscale: Contrast = 1:1:3:3.

#### Network layers selection

**Fig. 8.**
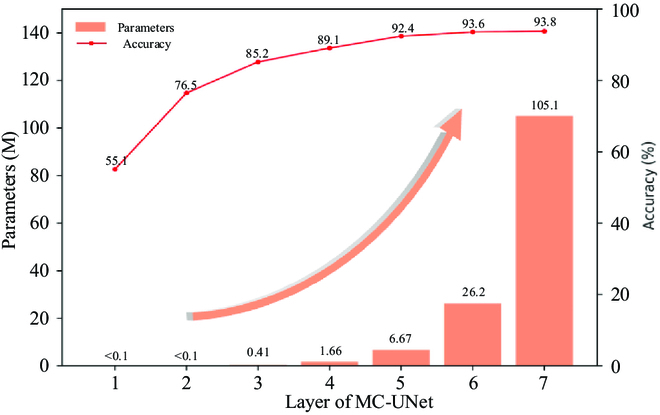
Comparison experiment of MC-UNet network layers.

A network that is excessively shallow will result in insufficient network model fitting, resulting in a poor segmentation effect. A network that is excessively deep might cause the network to perform well. However, it has a variety of serious flaws: large network model, long segmentation time, and so on. We should achieve a balance between precision and efficiency. We compared the layers of the backbone network UNet from level 1 to 7 using the MC-UNet architecture, and the results are shown in Fig. 8. At layers first-fifth, as the network depth grows, so does the Accuracy, and the variance between them all exceeds 3%. However, above the fifth floor, the growth rate dropped dramatically, especially on the sixth and seventh floors, where the variation is just 0.2%. As the model deepens, the number of network parameters continues to increase, and the rate of expansion of the parameter number accelerates. Considering the experimental results, we selected MC-UNet with a 5-layer UNet network as the backbone as the segmentation network model for tomato leaf disease.

#### Effectiveness analysis of individual modules

1. Effectiveness of MCM

The MCM module is primarily composed of 3 groups of convolution and SE Attention. We studied the 2 components individually to ensure their efficacy.

The design of the convolution kernel is closely related to feature extraction. A single convolution kernel can only be used for feature extraction in special environment. Too many convolution kernels will significantly increase the network’s burden, resulting in larger network parameters and longer operation time. To determine the optimal situation, we compare the performance of various convolution kernel scales on MCM. The experimental findings are shown in Table 5.

**Table 5.** Experimental results for different kernel combinations.

Kernel combination	mAP	FPS
3 × 3	0.8621	15.86
5 × 5	0.8709	15.69
7 × 7	0.8505	15.57
3 × 3, 5 × 5	0.8801	13.31
3 × 3, 7 × 7	0.8730	12.88
5 × 5, 7 × 7	0.8731	12.47
3 × 3, 5 × 5, 7 × 7	0.8842	10.12
3 × 3, 5 × 5, 7 × 7, 9 × 9	0.8891	8.74
3 × 3, 5 × 5, 7 × 7, 9 × 9, 11 × 11	0.8871	7.21

The network’s mAP achieves 88.42% when 3 convolution kernels are used. The FPS reduces (−1.38, −2.91) as the number of convolution kernels rises, while the effect is restricted (+0.49%, +0.29%). The network has been basically fitted, and excessive convolution kernels cannot enhance the network.

SE Module is used to extract edge information. We compare CBAM Attention [48], Coordinate Attention [49], and Efficient Channel Attention [50] in MCM. The experimental findings are shown in Table 6.

**Table 6.** Exploring the best Attention mechanism.

Method	mAP	FPS
Without Attention	0.8691	10.98
CBAM Attention	0.8727	10.54
Coordinate Attention	0.8867	9.91
Efficient Channel Attention	0.8804	10.13
SE Module	0.8842	10.15

Coordinate Attention has the highest mAP, but it also has the lowest FPS. Using each model, the accuracy of the network was improved. This indicates that the use of various attentions is helpful for segmentation of tomato leaf diseases. Combining mAP and FPS metrics, SE Module was introduced to enhance the model segmentation ability.

2. Effectiveness of CAFM

Gating structure has previously been applied to CAFM, improving the extraction of tiny diseases by feature-enhanced multiscale output fusion at each scale. The gating structure concentrates on providing gating signals via feature relationships in order to highlight features. The UNet Backbone has skip connections, causing Encode and Decode of the same dimension to combine. In this section, we will evaluate the performance of CAFM output fusion in the Decode layer, as well as Encode and Decode fusion output through skip connection. The testing results are shown in Table 7.

**Table 7.** Comparison test of Gated mechanism.

Method	mAP	Parameters	FPS
Without CAFM	0.8497	14.89M	11.97
Only with Decode output	0.8842	6.67M	10.12
Only with Skip-connection output	0.8699	11.72M	9.54
Two ablations	0.8808	6.65M	9.98

The model works well when the outputs are fused using CAFM at the Decode layer. Encode output improves the performance of the model in the fusion process, although the effect is not as pronounced as when Decode output is employed. It is worth noting that the results of CAFM applied to both sections simultaneously are lower in mAP and FPS than the output of the Decode component. This is because the preceding Encode and Decode fusion results interfere with the input features in the Decode layer, resulting in inaccurate feature weight extraction. In conclusion, applying CAFM on the model’s output fusion of the Decode layer is the efficient strategy.

3. Effectiveness of SoftPool

We employ AveragePool, RandPool, and MaxPool to test the network’s segmentation efficacy in order to evaluate the effectiveness of SoftPool and determine the pooling strategy to maintain tomato disease traits. Our test results are shown in Table 8.

**Table 8.** Performance of different pooling functions.

Method	mAP	FPS
AveragePool	0.8721	11.31
RandPool	0.8601	12.42
MaxPool	0.8732	11.14
SoftPool	0.8842	11.04

The experimental results demonstrated that employing SoftPool as a pooling layer enhances the network’s mAP (+1.1%) when compared to UNet’s original MaxPool. As a result, SoftPool is utilized as the model’s maximum pooling layer.

#### Ablation experiment

**Fig. 9.**
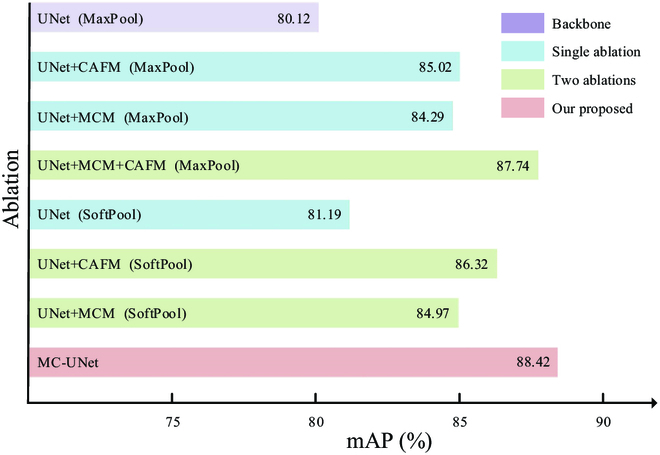
Ablation experiments of MC-UNet.

We undertake ablation experiments on the tomato disease image dataset using the proposed MC-UNet network to verify its efficacy. We employ the strategy of controlling factors to gradually add MCM and CAFM and selectively replace MaxPool with SoftPool to the standard UNet network as the backbone. We analyze the performance of each module and their importance to the network by analyzing mAP variations. Figure 9 shows the related experimental results.

According to the results of the experiments, the CAFM proposed in the paper instead of the traditional feature map output and the traditional skip connection achieved the best improvement effect, and the fusion improved by 2% to 4% under different conditions, suggesting that multiscale reflection of tomato leaves disease information is necessary. The use of SoftPool gives a smaller improvement to the network, about 1%. However, it is still an improvement. In conclusion, the addition of each module led to mAP’s improvement of the model. The above 8 sets of experiments prove that: our proposed 2 modules MCM and CAFM and the replacement of SoftPool are effective.

#### Operation process and result display of MC-UNet

**Fig. 10.**
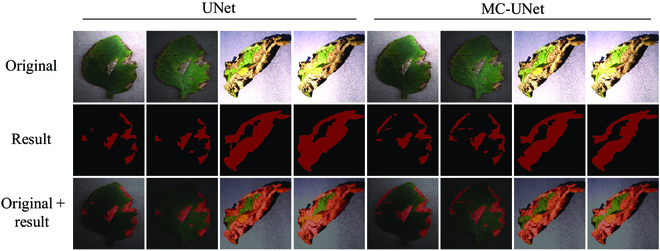
MC-UNet running results in different environments.

To visualize the robustness of MC-UNet in different lighting environments, we tested the network using a point light to simulate the actual environment. The corresponding results are shown in Fig. 10.

It can be seen that MC-UNet can be applied to different lighting environments, while the baseline network UNet segmentation is less effective. When the same target is under different illumination, UNet is more influenced by illumination and the segmentation results are inconsistent, while MC-UNet does not show obvious differences and has good robustness. This is attributed to the excellent perceptual ability of MC-UNet. Besides, for tiny and edge-blurred leaf diseases, MC-UNet significantly outperforms the baseline network.

**Fig. 11.**
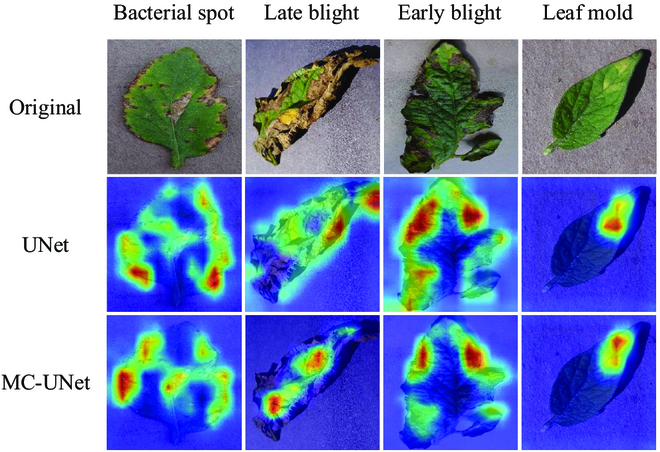
The effect of proposed MC-UNet.

In addition, we visualized the disease areas of interest to the MC-UNet network using the GradCAM, as shown in Fig. 11. Compared to UNet, MC-UNet are precisely focused on the disease areas. In Bacterial spot, the method in this paper accurately senses all disease areas, even the smaller ones. In Early blight, both networks sense the edge disease, but UNet segments part of the ground shade, which is not as accurate and reliable as the method in this paper. In summary, MC-UNet performs well on the tomato leaf disease segmentation task, especially in tiny and edge diseases.

### Experiment comparing MC-UNet with other models

**Fig. 12.**
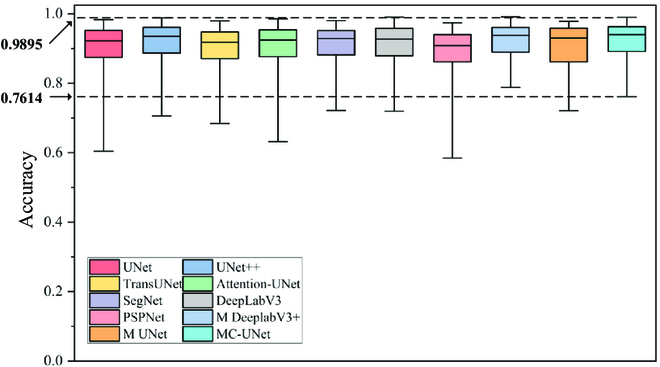
Boxplot about MC-UNet and other comparison methods.

In this experiment, the evaluation index defined in Evaluation indicators was used to test the performance of the MC-UNet model with that of UNet, UNet++, TransUNet [51], Attention-UNet [52], SegNet [53], DeepLabV3 [54], PSPNet [55], M DeeplabV3+ [56] and M UNet [57]. Accuracy distribution of each model is plotted using Boxplot, as shown in Table 9 and Fig. 12.

**Table 9.** Comparison of the main performance of different methods.

Model	mAP	Dice	Parameters	P	Cohen's *d*	FPS
UNet	0.8777	0.6431	24.89M	0.956	0.177	11.97
UNet++	0.8821	0.6503	32.02M	0.960	0.080	9.22
TransUNet	0.8537	0.6429	41.81M	0.945	0.201	7.31
Attention-UNet	0.8715	0.6482	14.85M	0.950	0.124	9.97
SegNet	0.8714	0.6272	14.86M	0.941	0.145	15.34
DeepLabV3	0.8611	0.6340	41.29M	0.940	0.187	8.79
PSPNet	0.8149	0.5298	6.92M	0.814	0.355	18.62
M DeeplabV3+	0.8793	0.6435	19.23M	0.959	0.080	7.88
M UNet	0.8648	0.6201	12.47M	0.923	0.107	12.30
MC-UNet	0.8842	0.6482	6.67M	0.962	0.064	10.12

In terms of Accuracy metrics, UNet++ and SegNet, in addition to our model, perform well in segmentation-based models: UNet (90.42%) is somewhat higher than SegNet’s (90.17%). DeepLabV3 also performs well (90.03%), but PSPNet performs the weakest of all models (88.65%). Despite the challenges, certain segmentation-based models (UNet and UNet++) outperform. One reason for this advantage might be due to the segmentation process. In fact, during the upsampling process, the U-Net with the skip connection structure added the feature map of the symmetrical layer on the channel, realizing the information fusion of low-level features and high-level features, so that the network can extract and retain more local details of tomato disease information, thereby improving image segmentation accuracy. Based on this, it is advisable to choose UNet as the primary network. However, the number of parameters in the TransUNet network is enormous, and the outcome is poor. This is owing to its unique Transformer structure, which is connected to all locations depending on each part’s prediction. It has extremely high hardware requirements for image segmentation tasks based on leaf diseases. In comparison to UNet and UNet++, our model achieves a greater segmentation efficiency while requiring fewer model parameters.

In the Wilcoxon signed-rank test results, the *P* value represents whether model output and labeled results are significant. If significant (*P* < 0.05), there was a big difference between the segmentation results of the model and the actual disease area. Cohen's *d* value represents the difference effect size, and the lower the value, the smaller the difference amplitude. It can be seen from the table that MC-UNet has the highest *P* value and Cohen's *d* is the lowest. This indicated that it performed optimally in the segmentation performance of tomato leaf diseases. However, when compared to other networks, the MC-UNet network model’s FPS score is somewhat low. This means that it processes fewer images per second but basically meets the requirements of intellectual agriculture.

In Fig. 12, we show the performance of MC-UNet and other networks on Accuracy in the form of Boxplot. As can be seen from the figure, MC-UNet, like all networks, also has a poor segment result (0.7614), but on average, MC-UNet performs well, outperforming other networks in the upper quartile, midpoint, and lower quartile.

**Fig. 13.**
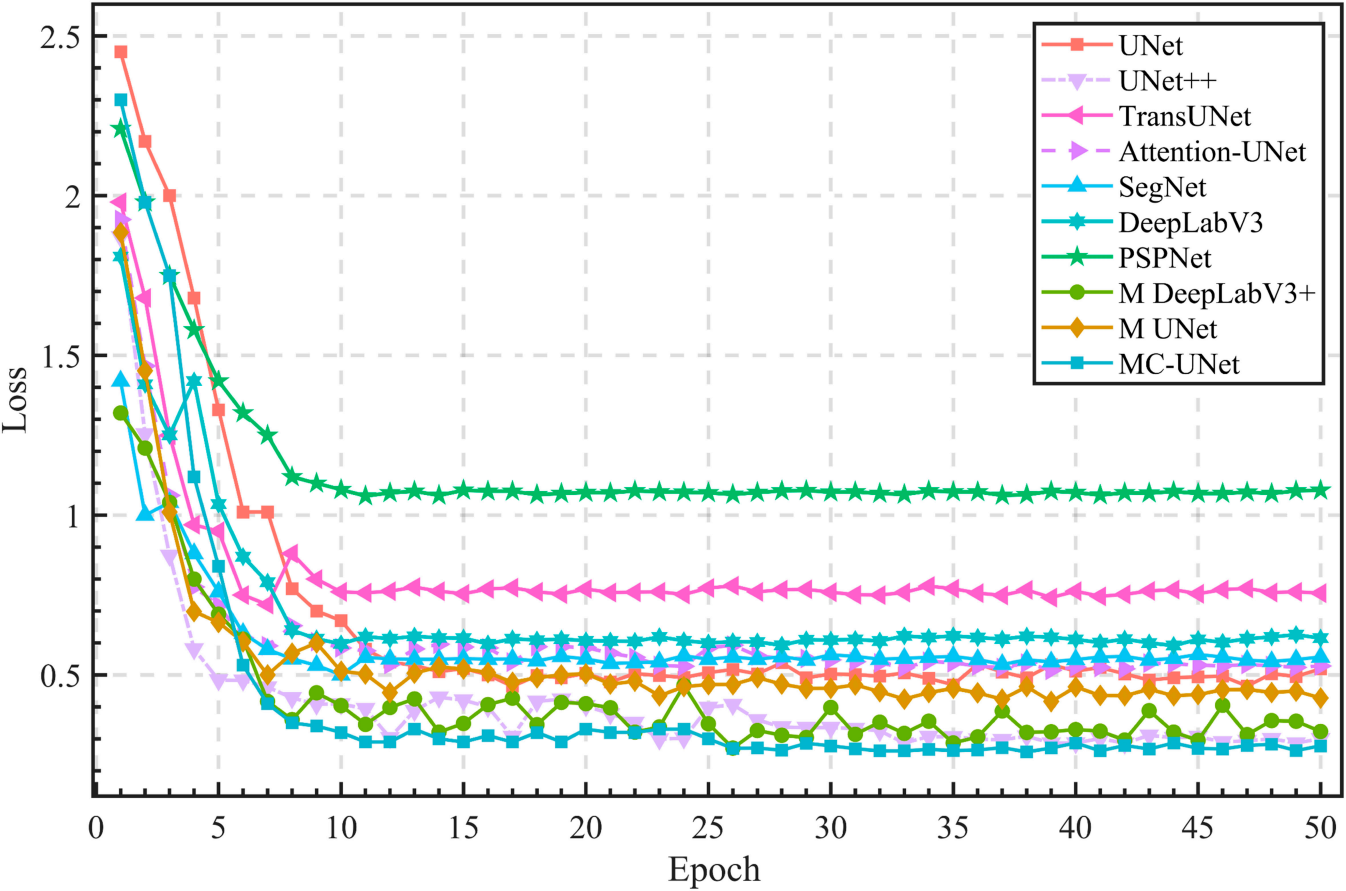
Loss curves of the MC-UNet and other methods.

The curve of loss values of different segmentation models changing with epoch is shown in Fig. 13. As can be seen from the figure, the loss value of different models varies greatly within the first few epochs, but the difference becomes smaller after 10 epochs. This shows that the network model tends to converge after a period of training. Of the remaining models, only UNet++ had a slightly lower loss than MC-UNet (about 0.2), while PSPNet had the highest loss value (about 1.0). The convergence rate of MC-UNet is faster in all networks, and the loss value is relatively low (about 0.25). This reflects our network has a strong generalization ability.

**Fig. 14.**
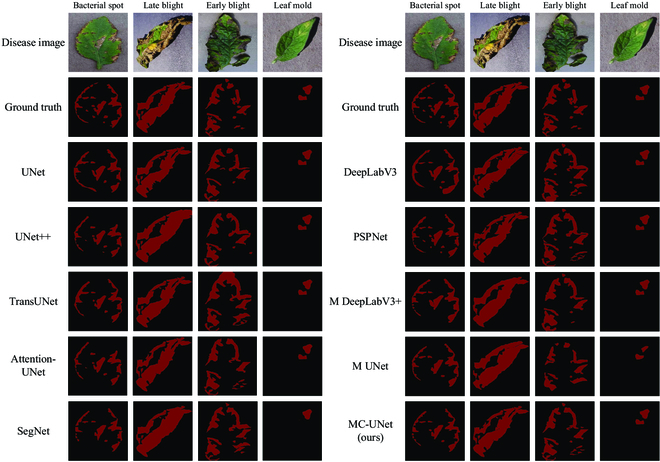
The effect of running various methods.

In order to visually compare the differences in the segmentation effects of various networks, we selected 4 images of each disease for visualization. Figure 14 shows the original disease image, ground truth, and segmentation results.

In Bacterial spot, some of the disease is very small and at the edge of the leaf. Some networks (such as UNet, Attention-UNet, and SegNet) segment the edge region of the leaf incorrectly, while MC-UNet segmentation of the disease is basically consistent with the actual situation. This indicates that the feature map generated by MCM is transmitted to the decoding layer through downsampling and skip connection, which enables the network to better learn the features of tiny diseases. Compared with other networks, the segmentation result of MC-UNet proves the validity of the module.

In Late blight, edge disease is mixed with shadow. Compared with other networks such as UNet, MC-UNet can distinguish the disease site and shadow area well. Even if the disease area is more complex, the network can be segmented normally. In addition, for the shadow on the leaf, the network can also distinguish the actual disease region by the semantic information of the neighboring areas. This is because the CAFM combines the characteristic information of each layer, enabling the network to perceive the disease from different levels and enhancing the ability of the network to distinguish irrelevant areas.

In Early blight and Leaf mold, the segmentation effect of each network is comparable. However, for some disease areas, the segmentation results of each network are poor. In addition, because some edge diseases are difficult to define and label, MC-UNet cannot learn their characteristics normally, resulting in a certain deviation in the segmentation process.

In summary, MC-UNet is a more suitable model for tomato leaf disease segmentation compared to other networks.

## Discussion

**Fig. 15.**
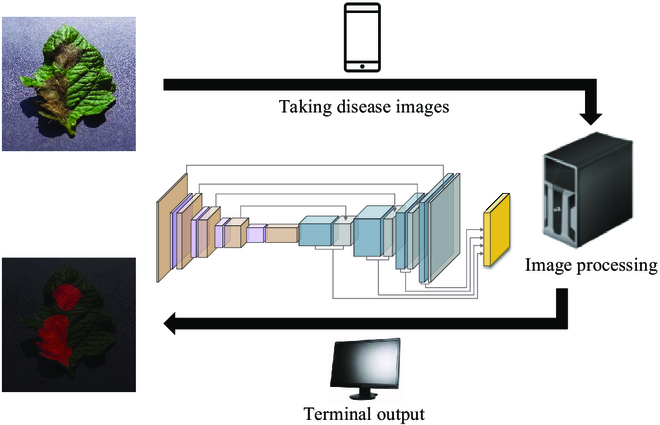
Schematic diagram of the MC-UNet-based automatic disease assessment system.

To test the effectiveness of MC-UNet model for tomato leaf disease segmentation, we built an automatic disease assessment system. Figure 15 depicts the architecture of the system. First, we took pictures with the cell phone (Huawei Mate10) to acquire disease images and then sent the acquired images to the image processing module. The image-processing module refers to segmentation of the captured image data using the MC-UNet model. To match the model, the image size will be converted automatically to 256 × 256. Finally, the input images and analysis results would be displayed through the terminal, and specific treatment recommendations were given. When the system is deployed, tomato disease images will be sent to the server for detection via the network, and the results will be fed back in a timely manner to help in the specific analysis and evaluation of tomato diseases.

**Fig. 16.**
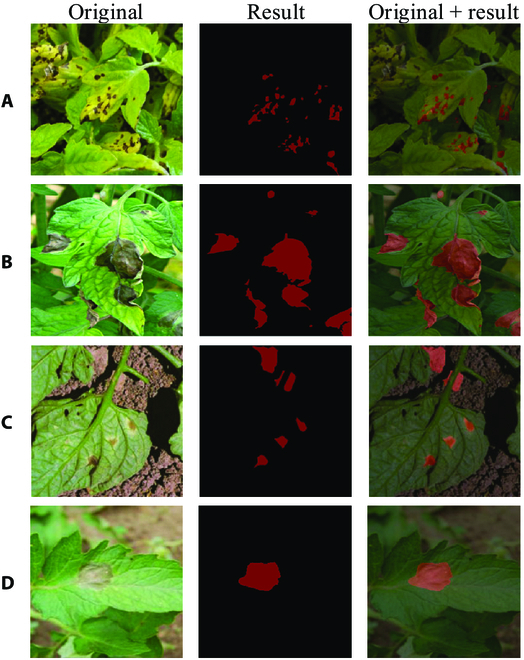
Real applications. (A to D) Different environment and disease distributions.

The images based on the automated tomato disease detection were collected manually. A total of 107 tomato leaf disease images were captured at the vegetable base of Hunan Plant Protection Research Institute in June, and Fig. 16 shows some of the outputs of the evaluation system. The output results generally met our expectations and proved that our system is capable of performing the disease monitoring task. However, a small number of incorrect segmentations still occurred, as shown in Fig. 16C. MC-UNet incorrectly segmented the soil in the background section as the disease site. This is because under real conditions, the soil is similar to some key disease features. Since most of MC-UNet's training data are taken in simple backgrounds, segmentation in such complex backgrounds is not effective.

The current work also usually does not focus on disease images in complex backgrounds. For example, in the dataset captured by Zhang et al. [58], the leaf region occupied the majority of the image. This allowed the network to focus on the characteristics of the disease. Barbed [59] removed the background from the images and found that the predictive power of the network was improved. To address the problem of complex backgrounds, we consider using a multistage segmentation model in future work. Also, we will collect as many tomato disease leaf images with complex backgrounds as possible to enhance the model's resistance to interference.

We proposed an approach based on MC-UNet to segment tomato leaf disease with tiny targets and blurred edges. Here are our conclusions:

1. Image augmentation experiments show that augmenting the dataset using the methods of brightness and contrast improves model segmentation accuracy significantly. Compared with the original dataset, the augmented dataset effectively improved the model's ability to adapt to different tomato leaf disease images.

2. The module analysis experiment shows that the CAFM improves the mAP value of the segmentation model with a small number of parameters, while MCM's convolution kernel and SE Attention comprehensively consider segmentation effect and performance.

3. When compared to traditional and advanced segmentation networks, our proposed model has a parameter size of 6.67M, an accuracy of 91.32%, and a mAP of 88.42%. Our model outperforms others. It can be efficiently applied to the segmentation task of tomato leaf disease to improve disease control.

The model proposed in this paper has good adaptability to the task of tomato leaf disease segmentation, but it does not perform well under complex background. In the future, we will specialize in dealing with complex background. More complicated background photos will also be gathered and inputted to train the model. We hope that the research results of this paper can provide a reference for other work and promote the development of intellectual agriculture.

## Data Availability

Some of the datasets that were used and analyzed in this study have been uploaded to the website https://github.com/ZhouGuoXiong/MC-UNet. In addition, all the homemade datasets in this study (6,372 sheets in total) can be obtained by contacting the corresponding author.
